# 1-Phenyl-3*H*-2,3-benzodiazepin-4(5*H*)-one

**DOI:** 10.1107/S1600536812031327

**Published:** 2012-07-14

**Authors:** Ballo Daouda, Frédéric Bihel, Mouhamadou Lamine Doumbia, El Mokhtar Essassi, Seik Weng Ng

**Affiliations:** a Laboratoire de Chimie Organique Hétérocyclique, Pôle de Compétences Pharmacochimie, Université Mohammed V-Agdal, BP 1014 Avenue Ibn Batout, Rabat, Morocco; b Laboratoire d’Innovation Thérapeutique UMR CNRS/UdS 7200, Faculté de Pharmacie de Strasbourg, 74 route du Rhin, BP 24 67401 ILLKIRCH Cedex, France; c Department of Chemistry, University of Malaya, 50603 Kuala Lumpur, Malaysia; d Chemistry Department, King Abdulaziz University, PO Box 80203, Jeddah, Saudi Arabia

## Abstract

The seven-membered ring in the title compound, C_15_H_12_N_2_O, adopts a boat-shaped conformation (with the methyl­ene C atom as the prow and the double-bond C=N pair of atoms as the stern). In the crystal, adjacent mol­ecules are linked by an N—H⋯O hydrogen bond to generate helical chains running along the *a* axis of the ortho­rhom­bic unit cell.

## Related literature

For the synthesis and pharmacological properties of the title compound, see: Flammang & Wermuth (1976 ▶); Wermuth & Flammang (1971 ▶). For related structures, see: Bruno *et al.* (2001 ▶, 2003 ▶).
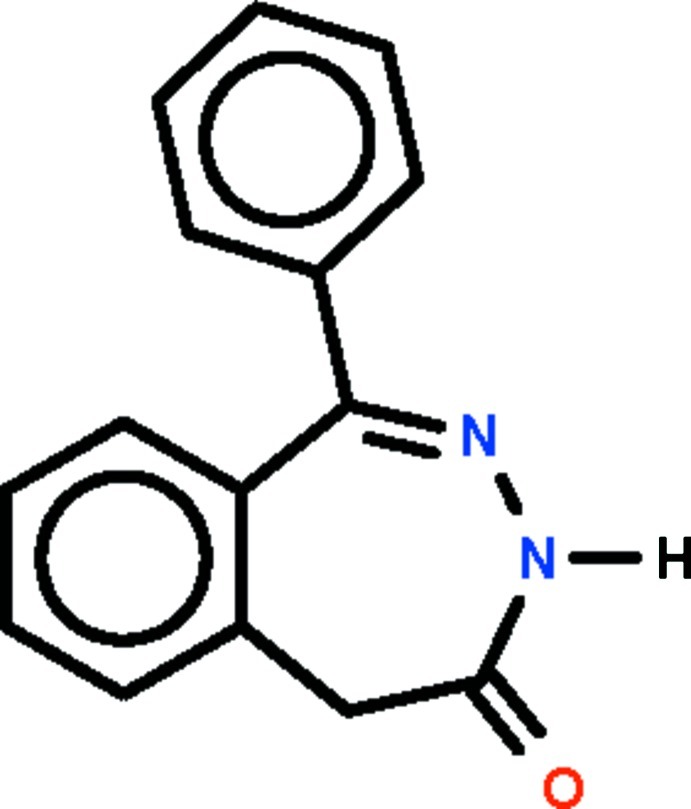


## Experimental

### 

#### Crystal data

C_15_H_12_N_2_O
*M* *_r_* = 236.27
Orthorhombic, 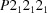

*a* = 5.4718 (1) Å
*b* = 8.4020 (1) Å
*c* = 26.3250 (5) Å
*V* = 1210.27 (4) Å^3^
*Z* = 4
Mo *K*α radiation
μ = 0.08 mm^−1^
*T* = 293 K
0.23 × 0.20 × 0.17 mm

#### Data collection

Bruker APEX DUO CCD diffractometer
9472 measured reflections
2063 independent reflections
1899 reflections with *I* > 2σ(*I*)
*R*_int_ = 0.021

#### Refinement

*R*[*F* ^2^ > 2σ(*F* ^2^)] = 0.040
*wR*(*F* ^2^) = 0.115
*S* = 1.04
2063 reflections
211 parameters
All H-atom parameters refined
Δρ_max_ = 0.27 e Å^−3^
Δρ_min_ = −0.17 e Å^−3^

### 

Data collection: *APEX2* (Bruker, 2010 ▶); cell refinement: *SAINT* (Bruker, 2010 ▶); data reduction: *SAINT*; program(s) used to solve structure: *SHELXS97* (Sheldrick, 2008 ▶); program(s) used to refine structure: *SHELXL97* (Sheldrick, 2008 ▶); molecular graphics: *X-SEED* (Barbour, 2001 ▶); software used to prepare material for publication: *publCIF* (Westrip, 2010 ▶).

## Supplementary Material

Crystal structure: contains datablock(s) global, I. DOI: 10.1107/S1600536812031327/zs2221sup1.cif

Structure factors: contains datablock(s) I. DOI: 10.1107/S1600536812031327/zs2221Isup2.hkl

Supplementary material file. DOI: 10.1107/S1600536812031327/zs2221Isup3.cml

Additional supplementary materials:  crystallographic information; 3D view; checkCIF report

## Figures and Tables

**Table 1:** Hydrogen-bond geometry (Å, °)

*D*—H⋯*A*	*D*—H	H⋯*A*	*D*⋯*A*	*D*—H⋯*A*
N2—H2⋯O1^i^	0.90 (3)	1.92 (3)	2.812 (2)	176 (2)

Symmetry code: (i) 
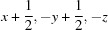
.

**Fig. 1.**
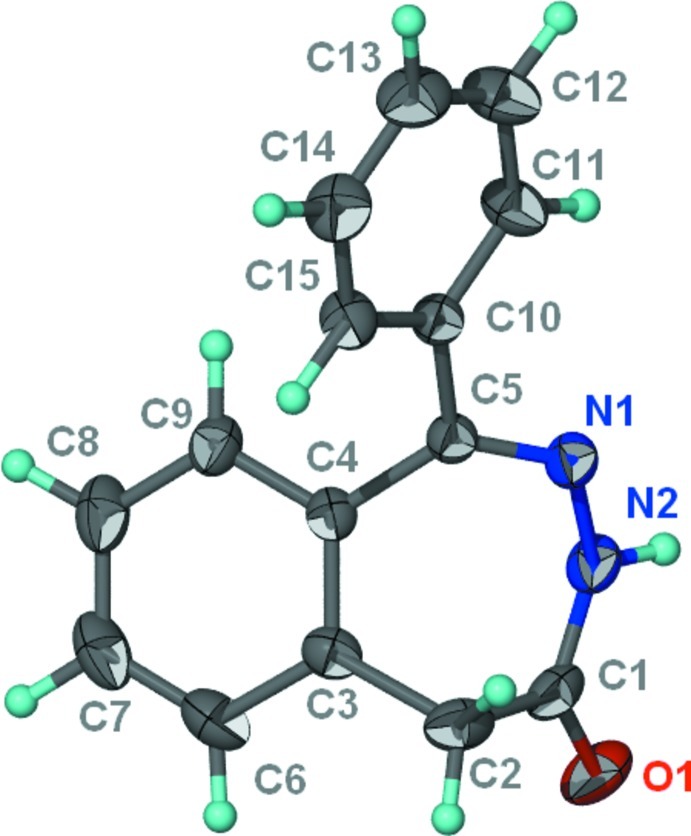
Thermal ellipsoid plot (Barbour, 2001) of C15H12N2O2 at the 50% probability level.

**Fig. 2.**
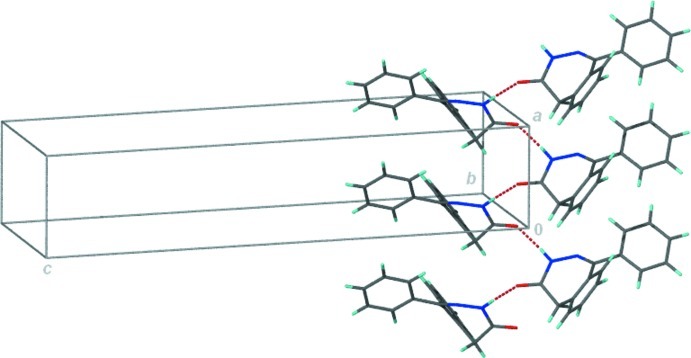
Hydrogen-bonded chain motif.

## supplementary crystallographic information

### Comment

The benzodiazepinone homolog, C_15_H_12_N_2_O (Scheme I), is a pharmacological compound exhibiting tranquilizer activity; its crystal structure has not previously been reported. When the benzene ring that is fused with the seven-membered ring carries a dioxolo substituent, the compound exists as a centrosymmetric dimer that is held together by an N—H···N hydrogen bond [3.030 (3) Å] (Bruno *et al.*, 2003). In contrast, with a pair of methoxy substituents, the compound is also a centrosymmetric dimer but the two halves are held together by an N—H···O hydrogen bond [2.876 (2) Å] (Bruno *et al.*, 2001).

The seven-membered ring in C_15_H_12_N_2_O adopts a boat-shaped conformation (Fig. 1). Adjacent molecules are linked by an N—H···O hydrogen bond (Table 1) to generate one-dimensional helical chains running along the *a*-axis of the orthorhombic unit cell (Fig. 2).

### Experimental

Ethoxycarbonylmethyl-2-benzophenone (1.34 g, 5 mmol) was heated with hydrazine hydrate (0.50 g, 10 mmol) in ethanol (30 ml) for 3 hours with the progress of the reaction monitored by thin layer chromatography. The solvent was removed and the white powder was recrystallized from ethanol to affford colorless crystals. The procedure was that reported in the literature (Flammang & Wermuth, 1976; Wermuth & Flammang, 1971).

### Refinement

Hydrogen atoms were freely refined. The (0 0 2) reflection was omitted owing to bad disagreement. In the absence of heavy atoms, 1461 Friedel pairs were merged.

### Crystal data

**Table:**

C_15_H_12_N_2_O	*F*(000) = 496
*M**_r_* = 236.27	*D*_x_ = 1.297 Mg m^−^^3^
Orthorhombic, *P*2_1_2_1_2_1_	Mo *K*α radiation, λ = 0.71073 Å
Hall symbol: P 2ac 2ab	Cell parameters from 4920 reflections
*a* = 5.4718 (1) Å	θ = 2.9–32.7°
*b* = 8.4020 (1) Å	µ = 0.08 mm^−^^1^
*c* = 26.3250 (5) Å	*T* = 293 K
*V* = 1210.27 (4) Å^3^	Prism, colorless
*Z* = 4	0.23 × 0.20 × 0.17 mm

### Data collection

**Table:**

Bruker APEX DUO CCD diffractometer	1899 reflections with *I* > 2σ(*I*)
Radiation source: fine-focus sealed tube	*R*_int_ = 0.021
Graphite monochromator	θ_max_ = 30.0°, θ_min_ = 2.9°
ω scans	*h* = −7→7
9472 measured reflections	*k* = −10→11
2063 independent reflections	*l* = −37→35

### Refinement

**Table:**

Refinement on *F*^2^	Primary atom site location: structure-invariant direct methods
Least-squares matrix: full	Secondary atom site location: difference Fourier map
*R*[*F*^2^ > 2σ(*F*^2^)] = 0.040	Hydrogen site location: inferred from neighbouring sites
*wR*(*F*^2^) = 0.115	All H-atom parameters refined
*S* = 1.04	*w* = 1/[σ^2^(*F*_o_^2^) + (0.0814*P*)^2^ + 0.0803*P*] where *P* = (*F*_o_^2^ + 2*F*_c_^2^)/3
2063 reflections	(Δ/σ)_max_ = 0.001
211 parameters	Δρ_max_ = 0.27 e Å^−^^3^
0 restraints	Δρ_min_ = −0.17 e Å^−^^3^

### Fractional atomic coordinates and isotropic or equivalent isotropic displacement parameters (Å^2^)

**Table:**

	*x*	*y*	*z*	*U*_iso_*/*U*_eq_	
O1	−0.1095 (3)	0.36819 (18)	−0.01027 (5)	0.0604 (4)	
N1	0.1515 (3)	0.37477 (15)	0.11324 (4)	0.0380 (3)	
N2	0.1083 (3)	0.36162 (16)	0.06118 (5)	0.0413 (3)	
C1	−0.0928 (3)	0.40531 (19)	0.03479 (6)	0.0424 (3)	
C2	−0.2805 (3)	0.5014 (2)	0.06309 (7)	0.0477 (4)	
C3	−0.1668 (3)	0.6578 (2)	0.07707 (5)	0.0382 (3)	
C4	0.0289 (3)	0.65874 (16)	0.11127 (5)	0.0329 (3)	
C5	0.1167 (3)	0.51028 (16)	0.13530 (5)	0.0319 (3)	
C6	−0.2431 (4)	0.8013 (3)	0.05550 (7)	0.0520 (4)	
C7	−0.1259 (5)	0.9415 (2)	0.06675 (7)	0.0582 (5)	
C8	0.0708 (5)	0.9426 (2)	0.09960 (7)	0.0545 (5)	
C9	0.1462 (4)	0.80259 (19)	0.12240 (6)	0.0420 (4)	
C10	0.1850 (3)	0.51417 (17)	0.19011 (5)	0.0336 (3)	
C11	0.3855 (3)	0.4275 (2)	0.20722 (6)	0.0468 (4)	
C12	0.4501 (4)	0.4305 (3)	0.25822 (7)	0.0570 (5)	
C13	0.3130 (4)	0.5173 (3)	0.29244 (6)	0.0555 (5)	
C14	0.1132 (4)	0.6027 (2)	0.27610 (6)	0.0514 (4)	
C15	0.0503 (3)	0.6029 (2)	0.22465 (6)	0.0419 (3)	
H2	0.201 (5)	0.287 (3)	0.0465 (9)	0.057 (6)*	
H21	−0.325 (5)	0.439 (3)	0.0955 (8)	0.052 (6)*	
H22	−0.422 (6)	0.513 (3)	0.0425 (10)	0.074 (8)*	
H6	−0.385 (5)	0.794 (3)	0.0333 (10)	0.072 (8)*	
H7	−0.183 (6)	1.042 (4)	0.0503 (10)	0.082 (9)*	
H8	0.146 (5)	1.046 (3)	0.1093 (9)	0.063 (7)*	
H9	0.288 (4)	0.803 (3)	0.1463 (8)	0.051 (5)*	
H11	0.486 (6)	0.358 (3)	0.1827 (9)	0.067 (7)*	
H12	0.595 (5)	0.365 (3)	0.2686 (9)	0.057 (6)*	
H13	0.349 (6)	0.514 (4)	0.3290 (10)	0.087 (9)*	
H14	0.015 (6)	0.672 (3)	0.2998 (9)	0.065 (7)*	
H15	−0.088 (5)	0.666 (3)	0.2125 (8)	0.059 (6)*	

### Atomic displacement parameters (Å^2^)

**Table:**

	*U*^11^	*U*^22^	*U*^33^	*U*^12^	*U*^13^	*U*^23^
O1	0.0822 (9)	0.0608 (8)	0.0383 (6)	−0.0207 (8)	−0.0137 (6)	−0.0071 (5)
N1	0.0475 (7)	0.0350 (6)	0.0315 (5)	0.0012 (6)	−0.0012 (5)	−0.0018 (4)
N2	0.0526 (8)	0.0387 (6)	0.0328 (6)	−0.0001 (6)	−0.0006 (5)	−0.0077 (5)
C1	0.0505 (8)	0.0393 (7)	0.0374 (7)	−0.0179 (7)	−0.0053 (6)	−0.0008 (5)
C2	0.0350 (7)	0.0592 (10)	0.0490 (8)	−0.0102 (7)	−0.0059 (6)	0.0025 (8)
C3	0.0339 (7)	0.0451 (7)	0.0358 (6)	0.0030 (6)	0.0004 (5)	0.0026 (5)
C4	0.0374 (6)	0.0324 (6)	0.0289 (5)	0.0017 (6)	0.0023 (5)	0.0007 (5)
C5	0.0341 (6)	0.0320 (6)	0.0296 (5)	−0.0008 (5)	−0.0007 (5)	0.0005 (5)
C6	0.0492 (10)	0.0595 (10)	0.0474 (8)	0.0156 (9)	−0.0022 (8)	0.0102 (8)
C7	0.0819 (14)	0.0437 (9)	0.0490 (9)	0.0177 (10)	0.0068 (10)	0.0132 (7)
C8	0.0848 (14)	0.0341 (7)	0.0446 (8)	−0.0021 (9)	0.0065 (9)	0.0037 (6)
C9	0.0577 (10)	0.0346 (6)	0.0339 (6)	−0.0051 (7)	−0.0014 (7)	−0.0005 (5)
C10	0.0381 (7)	0.0336 (6)	0.0292 (5)	−0.0008 (6)	−0.0012 (5)	0.0015 (5)
C11	0.0477 (9)	0.0566 (9)	0.0362 (7)	0.0121 (8)	−0.0011 (6)	0.0046 (6)
C12	0.0551 (10)	0.0753 (13)	0.0408 (8)	0.0099 (11)	−0.0076 (7)	0.0116 (8)
C13	0.0683 (12)	0.0683 (11)	0.0300 (6)	−0.0056 (11)	−0.0064 (7)	0.0053 (7)
C14	0.0671 (11)	0.0554 (9)	0.0318 (7)	0.0003 (9)	0.0054 (7)	−0.0036 (7)
C15	0.0489 (8)	0.0424 (8)	0.0345 (6)	0.0050 (7)	0.0021 (6)	−0.0002 (6)

### Geometric parameters (Å, º)

**Table:**

O1—C1	1.2301 (19)	C7—C8	1.380 (3)
N1—C5	1.2922 (18)	C7—H7	1.00 (3)
N1—N2	1.3951 (16)	C8—C9	1.383 (2)
N2—C1	1.352 (2)	C8—H8	1.00 (3)
N2—H2	0.90 (3)	C9—H9	1.00 (2)
C1—C2	1.504 (3)	C10—C15	1.388 (2)
C2—C3	1.500 (3)	C10—C11	1.392 (2)
C2—H21	1.03 (2)	C11—C12	1.389 (2)
C2—H22	0.95 (3)	C11—H11	1.03 (3)
C3—C6	1.397 (2)	C12—C13	1.381 (3)
C3—C4	1.399 (2)	C12—H12	1.00 (3)
C4—C9	1.400 (2)	C13—C14	1.377 (3)
C4—C5	1.4790 (19)	C13—H13	0.98 (3)
C5—C10	1.4907 (17)	C14—C15	1.398 (2)
C6—C7	1.374 (3)	C14—H14	1.01 (3)
C6—H6	0.97 (3)	C15—H15	0.98 (3)
			
C5—N1—N2	119.10 (12)	C6—C7—H7	119.1 (18)
C1—N2—N1	128.39 (15)	C8—C7—H7	120.6 (18)
C1—N2—H2	115.5 (15)	C7—C8—C9	119.94 (18)
N1—N2—H2	112.5 (15)	C7—C8—H8	119.2 (16)
O1—C1—N2	119.15 (18)	C9—C8—H8	120.6 (16)
O1—C1—C2	124.27 (17)	C8—C9—C4	120.42 (16)
N2—C1—C2	116.56 (14)	C8—C9—H9	120.3 (14)
C3—C2—C1	107.98 (13)	C4—C9—H9	119.3 (14)
C3—C2—H21	109.8 (13)	C15—C10—C11	119.20 (14)
C1—C2—H21	107.4 (13)	C15—C10—C5	120.85 (13)
C3—C2—H22	112.8 (17)	C11—C10—C5	119.95 (13)
C1—C2—H22	109.2 (17)	C12—C11—C10	120.27 (16)
H21—C2—H22	109 (2)	C12—C11—H11	118.7 (15)
C6—C3—C4	119.03 (16)	C10—C11—H11	121.0 (15)
C6—C3—C2	122.19 (15)	C13—C12—C11	120.13 (18)
C4—C3—C2	118.73 (14)	C13—C12—H12	122.8 (13)
C3—C4—C9	119.40 (14)	C11—C12—H12	117.0 (14)
C3—C4—C5	121.27 (13)	C14—C13—C12	120.21 (15)
C9—C4—C5	119.32 (13)	C14—C13—H13	118.6 (19)
N1—C5—C4	126.78 (12)	C12—C13—H13	121.1 (19)
N1—C5—C10	114.67 (12)	C13—C14—C15	119.92 (17)
C4—C5—C10	118.49 (12)	C13—C14—H14	122.0 (15)
C7—C6—C3	120.88 (17)	C15—C14—H14	117.9 (15)
C7—C6—H6	123.6 (17)	C10—C15—C14	120.24 (16)
C3—C6—H6	115.5 (17)	C10—C15—H15	119.1 (13)
C6—C7—C8	120.30 (16)	C14—C15—H15	120.6 (13)
			
C5—N1—N2—C1	−51.1 (2)	C2—C3—C6—C7	−176.05 (19)
N1—N2—C1—O1	−171.86 (15)	C3—C6—C7—C8	0.2 (3)
N1—N2—C1—C2	9.7 (2)	C6—C7—C8—C9	−1.7 (3)
O1—C1—C2—C3	−113.71 (17)	C7—C8—C9—C4	1.8 (3)
N2—C1—C2—C3	64.68 (19)	C3—C4—C9—C8	−0.4 (2)
C1—C2—C3—C6	112.02 (18)	C5—C4—C9—C8	178.66 (15)
C1—C2—C3—C4	−65.26 (18)	N1—C5—C10—C15	−144.11 (15)
C6—C3—C4—C9	−1.1 (2)	C4—C5—C10—C15	38.6 (2)
C2—C3—C4—C9	176.25 (15)	N1—C5—C10—C11	35.6 (2)
C6—C3—C4—C5	179.85 (15)	C4—C5—C10—C11	−141.71 (16)
C2—C3—C4—C5	−2.8 (2)	C15—C10—C11—C12	−0.2 (3)
N2—N1—C5—C4	1.2 (2)	C5—C10—C11—C12	−179.95 (18)
N2—N1—C5—C10	−175.89 (13)	C10—C11—C12—C13	1.1 (3)
C3—C4—C5—N1	44.0 (2)	C11—C12—C13—C14	−0.7 (3)
C9—C4—C5—N1	−135.03 (17)	C12—C13—C14—C15	−0.6 (3)
C3—C4—C5—C10	−139.02 (14)	C11—C10—C15—C14	−1.1 (3)
C9—C4—C5—C10	41.9 (2)	C5—C10—C15—C14	178.62 (16)
C4—C3—C6—C7	1.2 (3)	C13—C14—C15—C10	1.5 (3)

### Hydrogen-bond geometry (Å, º)

**Table:**

*D*—H···*A*	*D*—H	H···*A*	*D*···*A*	*D*—H···*A*
N2—H2···O1^i^	0.90 (3)	1.92 (3)	2.812 (2)	176 (2)

Symmetry code: (i) *x*+1/2, −*y*+1/2, −*z*.
